# Uncovering potential genes in colorectal cancer based on integrated and DNA methylation analysis in the gene expression omnibus database

**DOI:** 10.1186/s12885-022-09185-0

**Published:** 2022-02-03

**Authors:** Guanglin Wang, Feifei Wang, Zesong Meng, Na Wang, Chaoxi Zhou, Juan Zhang, Lianmei Zhao, Guiying Wang, Baoen Shan

**Affiliations:** 1 The Second Department of Surgery, The Fourth Hospital of Hebei Medical University, Shijiazhuang, China; 2 Institute of Tumor, The Fourth Hospital of Hebei Medical University, Shijiazhuang, China; 3 Scientific Research Center, The Fourth Hospital of Hebei Medical University, No. 12, Jiankang Road, Chang’an District, Shijiazhuang, 050010 Hebei Province China; 4 Department of General Surgery, The Third Hospital of Hebei Medical University, Shijiazhuang, China

**Keywords:** Colorectal cancer, Differentially expressed genes, Differentially methylated genes, Diagnosis, Prognosis

## Abstract

**Background:**

Colorectal cancer (CRC) is major cancer-related death. The aim of this study was to identify differentially expressed and differentially methylated genes, contributing to explore the molecular mechanism of CRC.

**Methods:**

Firstly, the data of gene transcriptome and genome-wide DNA methylation expression were downloaded from the Gene Expression Omnibus database. Secondly, functional analysis of differentially expressed and differentially methylated genes was performed, followed by protein-protein interaction (PPI) analysis. Thirdly, the Cancer Genome Atlas (TCGA) dataset and in vitro experiment was used to validate the expression of selected differentially expressed and differentially methylated genes. Finally, diagnosis and prognosis analysis of selected differentially expressed and differentially methylated genes was performed.

**Results:**

Up to 1958 differentially expressed (1025 up-regulated and 993 down-regulated) genes and 858 differentially methylated (800 hypermethylated and 58 hypomethylated) genes were identified. Interestingly, some genes, such as *GFRA2* and *MDFI*, were differentially expressed-methylated genes. Purine metabolism (involved *IMPDH1*), cell adhesion molecules and PI3K-Akt signaling pathway were significantly enriched signaling pathways. *GFRA2*, *FOXQ1*, *CDH3*, *CLDN1*, *SCGN*, *BEST4*, *CXCL12*, *CA7*, *SHMT2*, *TRIP13*, *MDFI* and *IMPDH1* had a diagnostic value for CRC. In addition, *BEST4*, *SHMT2* and *TRIP13* were significantly associated with patients’ survival.

**Conclusions:**

The identified altered genes may be involved in tumorigenesis of CRC. In addition, *BEST4*, *SHMT2* and *TRIP13* may be considered as diagnosis and prognostic biomarkers for CRC patients.

**Supplementary Information:**

The online version contains supplementary material available at 10.1186/s12885-022-09185-0.

## Background

Colorectal cancer (CRC) is major cancer-related death [1, 2]. Sustained cell proliferation and invasion, enhanced angiogenesis and metastasis, and drug resistance are the major characteristics of CRC [3, 4]. Various factors are related to the development of CRC, such as genetics, polyposis, chronic inflammation, inflammatory bowel disease, increased body mass index, little physical activity, cigarette smoking, alcohol abuse and particular dietary habits [5–11]. Clinically, main curative treatments for CRC are radiotherapy, chemotherapy and surgical removal of lesions. The survival outcome of CRC patients is worse, with a 5-year survival rate of only 14.0% [12]. Therefore, it is important to understand the pathological mechanism of CRC.

Simons CCJM et al. found that the CpG island methylated phenotype is a major factor contributing to CRC carcinogenesis [13]. Furthermore, gene expression regulation by aberrant DNA methylation is extensively described for CRC. For example, abnormal methylation of septin 9 (*SEPT9*) is frequently reported in CRC, and the *SEPT9* methylation test has been used in early screening for CRC [14–16]. In order to further investigate the pathological mechanism of CRC, we performed both integrated analysis and DNA methylation analysis in the Gene Expression Omnibus database to find potential and valuable genes in CRC.

## Methods

### Datasets retrieval

We searched datasets from the GEO dataset with the keywords (Colorectal cancer) AND “*Homo sapiens*”[porgn:__txid9606]. All selected datasets were gene transcriptome and genome-wide DNA methylation expression data in the CRC tumor tissues and normal controls. Finally, a total of 3 datasets of gene transcriptome data (GSE113513, GSE87211 and GSE89076) and 2 datasets of genome-wide DNA methylation expression data (GSE101764 and GSE129364) were identified (Table 1). Clinical information of above datasets is shown in supplementary Table 1.

**Table 1:** Datasets of gene transcriptome data and genome-wide DNA methylation expression data in the GEO dataset

GEO accession	Author	Platform	Samples (N:P)	Year	Tissue
GSE113513	Jun Peng	GPL15207 [PrimeView] Affymetrix Human Gene Expression Array	14:14	2018	Colon and rectal tissue
GSE87211	Yue Hu	GPL13497 Agilent-026652 Whole Human Genome Microarray 4x44K v2 (Probe Name version)	160:203	2017	Rectal tissue
GSE89076	Kiyotoshi Satoh	GPL16699 Agilent-039494 SurePrint G3 Human GE v2 8x60K Microarray 039381 (Feature Number version)	39:41	2017	Colon and rectal tissue
GSE101764	Hauke Busch	GPL13534 Illumina HumanMethylation450 BeadChip (HumanMethylation450_15017482)	149:112	2017	Colon and rectal tissue
GSE129364	Yue Hu	GPL13534 Illumina HumanMethylation450 BeadChip (HumanMethylation450_15017482)	3:69	2019	Colon and rectal tissue

*N* normal controls, *P* patients with CRC

### Identification of differentially expressed and differentially methylated genes

Firstly, scale standardization was carried out for the common genes in 3 datasets of gene transcriptome data. The metaMA and limma packages were used to identify differentially expressed genes [17]. *P* values and effect sizes from data were calculated either from classical or moderated t-tests. These *p* values were combined by the inverse normal method. Benjamini hochberg threshold was used to calculate the false discovery rate (FDR). Finally, differentially expressed genes were obtained with the criterion of FDR and |Combined.effect size| ≥ 1.5. In addition, quantile standardization was performed for the common genes in 2 datasets of genome-wide DNA methylation expression data. Benjamini hochberg threshold was used to calculate the FDR. COHCAP package in R language was used to identify differentially methylated genes under the threshold of |Δβ| > 0.3 and FDR < 0.05.

### Functional analysis of differentially expressed and differentially methylated genes

To understand the function of differentially expressed and differentially methylated genes, we conducted Gene Ontology (GO) and the Kyoto Encyclopedia of Genes and Genomes (KEGG) analysis through David 6.8 (https://david.ncifcrf.gov/). FDR < 0.05 was considered as significant.

### PPI network

The BioGRID database was used to retrieve the predicted interactions between top 50 proteins and other proteins. In the network, node and edge represents protein and the interactions, respectively.

### Electronic and in vitro validation of differentially expressed and differentially methylated genes

The Cancer Genome Atlas (TCGA) dataset (involved 478 patients with CRC and 41 normal controls) was used to validate the expression of differentially expressed and differentially methylated genes. The expression result of these genes was shown by box plots.

In vitro validation QRT-PCR was also performed. The inclusion criteria of CRC patients was as follows: (1) Patients were diagnosed with CRC according to the pathological examination; (2) Patients underwent radical resection of CRC for the first time and received no chemoradiotherapy before; (3) patients had complete clinical data including medical history of present illness, personal history, family history, detailed physical examination data and postoperative pathological data. The exclusion criteria of CRC patients were as follows: (1) patients had other colorectal tumors, carcinoid, malignant melanoma, malignant lymphoma and so on; (2) patients had multiple primary CRC, familial adenomatous polyposis and concurrent or previous malignancy. According to the above criteria, 5 CRC patients were enrolled. Clinical information of these CRC patients was listed in Table 2. The tumor tissue and para-carcinoma tissue of these patients was collected. All participating individuals provided informed consent with the approval of the ethics committee of the local hospital. All the experimental protocol for involving humans was in accordance to guidelines of national/international/institutional or Declaration of Helsinki.

**Table 2:** The clinical information of CRC patients in the QRT-PCR

Number	Gender	Age	Tumor site	Maximum tumor diameter (cm)	Degree of tumor differentiation	TNM staging	Degree of intestinal wall invasion	Lymph node metastasis	Operation scheme
1	Male	57	Rectum	5	III, intermediate differentiation	T3N0M0	Fat	No	Laparoscopic radical resection of rectal cancer
2	Male	64	*Rectum*	6	II, intermediate differentiation	T3N0M0	Fat	No	Laparoscopic radical resection of rectal cancer
3	Female	64	Colon	4	II, intermediate differentiation	T4N0M0	Serous coat	No	Laparoscopic left hemicolectomy
4	Male	54	*Rectum*	4	II, intermediate differentiation	T3NOMO	Fat	No	Laparoscopic radical resection of rectal cancer
5	Female	61	*Rectum*	2.5	II, intermediate differentiation	T4N0M0	Serous coat	No	Laparoscopic radical resection of rectal cancer

Total RNA of the tissue and para-carcinoma tissue was extracted and synthesized DNA by FastQuant cDNA first strand synthesis kit (TIANGEN). Then real-time PCR was performed in the SuperReal PreMix Plus (SYBR Green) (TIANGEN). ACTB and GAPDH were used for internal reference. Relative mRNAs expression was analyzed by log2 (fold change) method.

### Diagnosis and prognosis analysis of differentially expressed and differentially methylated genes

We performed the ROC and survival analysis to assess the diagnostic and prognostic value of differentially expressed and differentially methylated genes in the TCGA dataset.

## Results

### Differentially expressed and differentially methylated genes in the GEO dataset

**Fig. 1:**
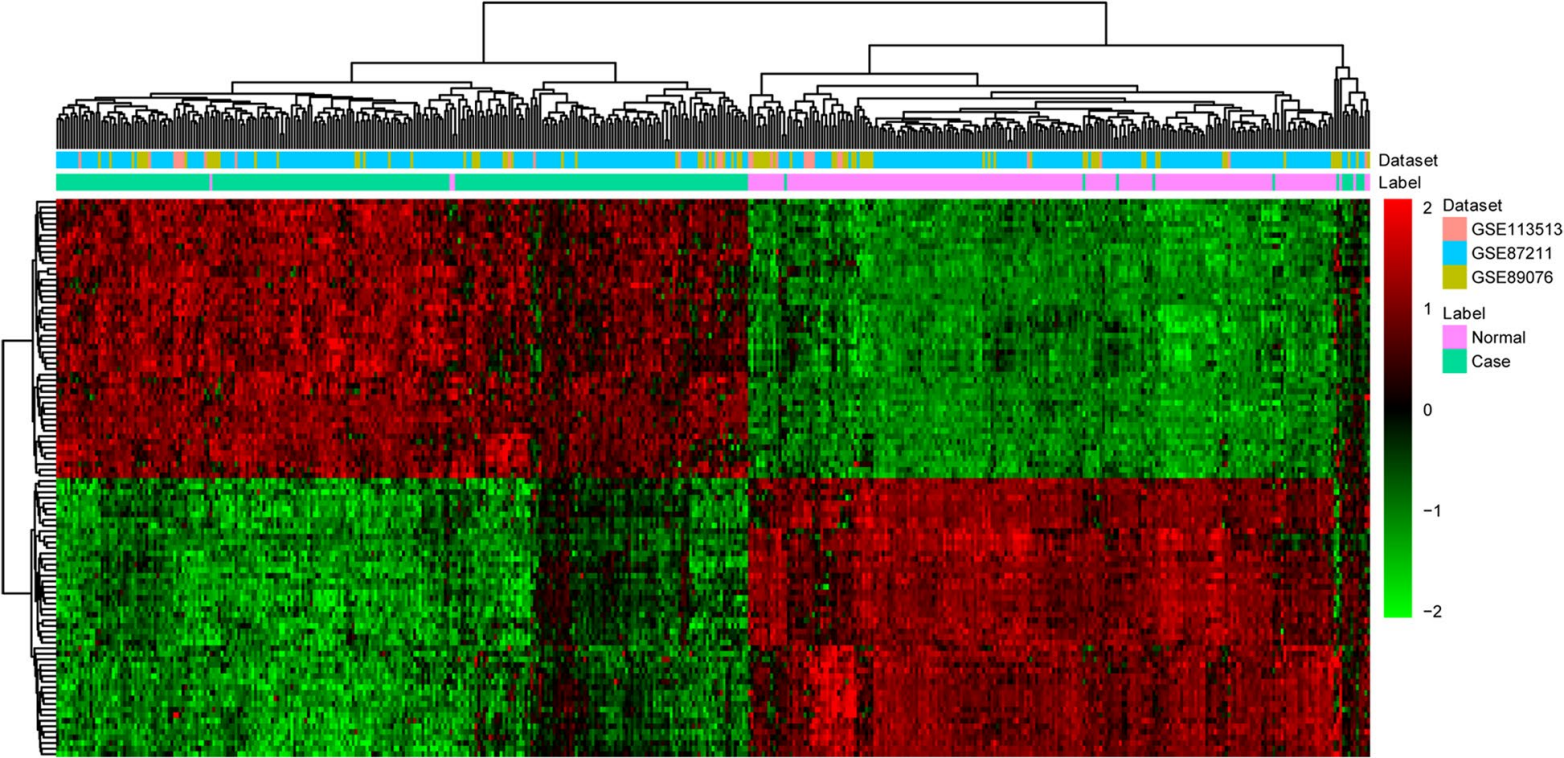
The heat map of top 100 differentially expressed genes in CRC. Diagram presents the result of a two-way hierarchical clustering of top 100 differentially expressed genes and samples. Each row and each column represents a differentially expressed gene and a sample, respectively

**Fig. 2:**
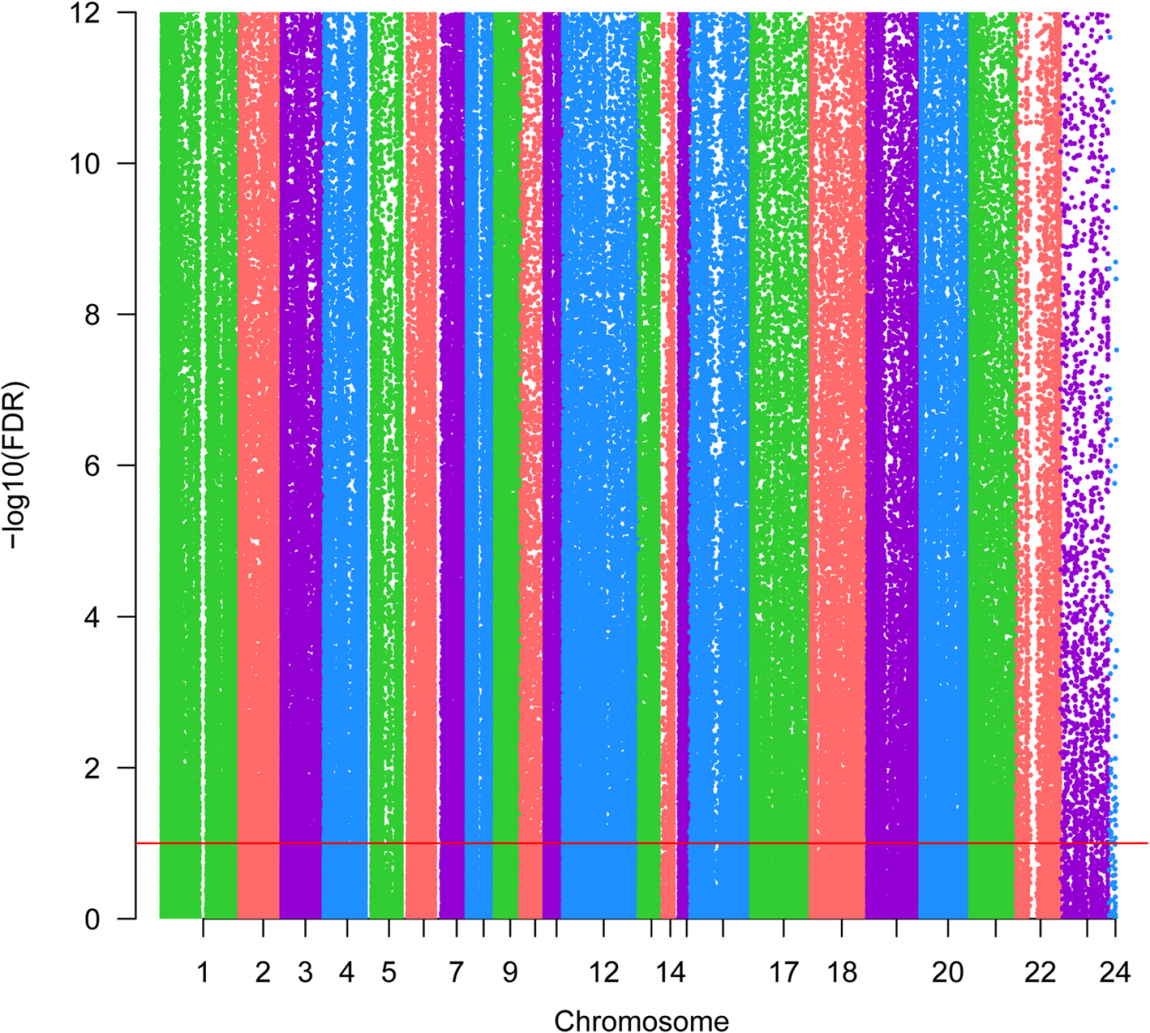
The Manhattan of all differential methylation sites in CRC. The x-axis represents the chromosome, the y-axis represents the -log10 (FDR) of differential methylation sites

**Fig. 3:**
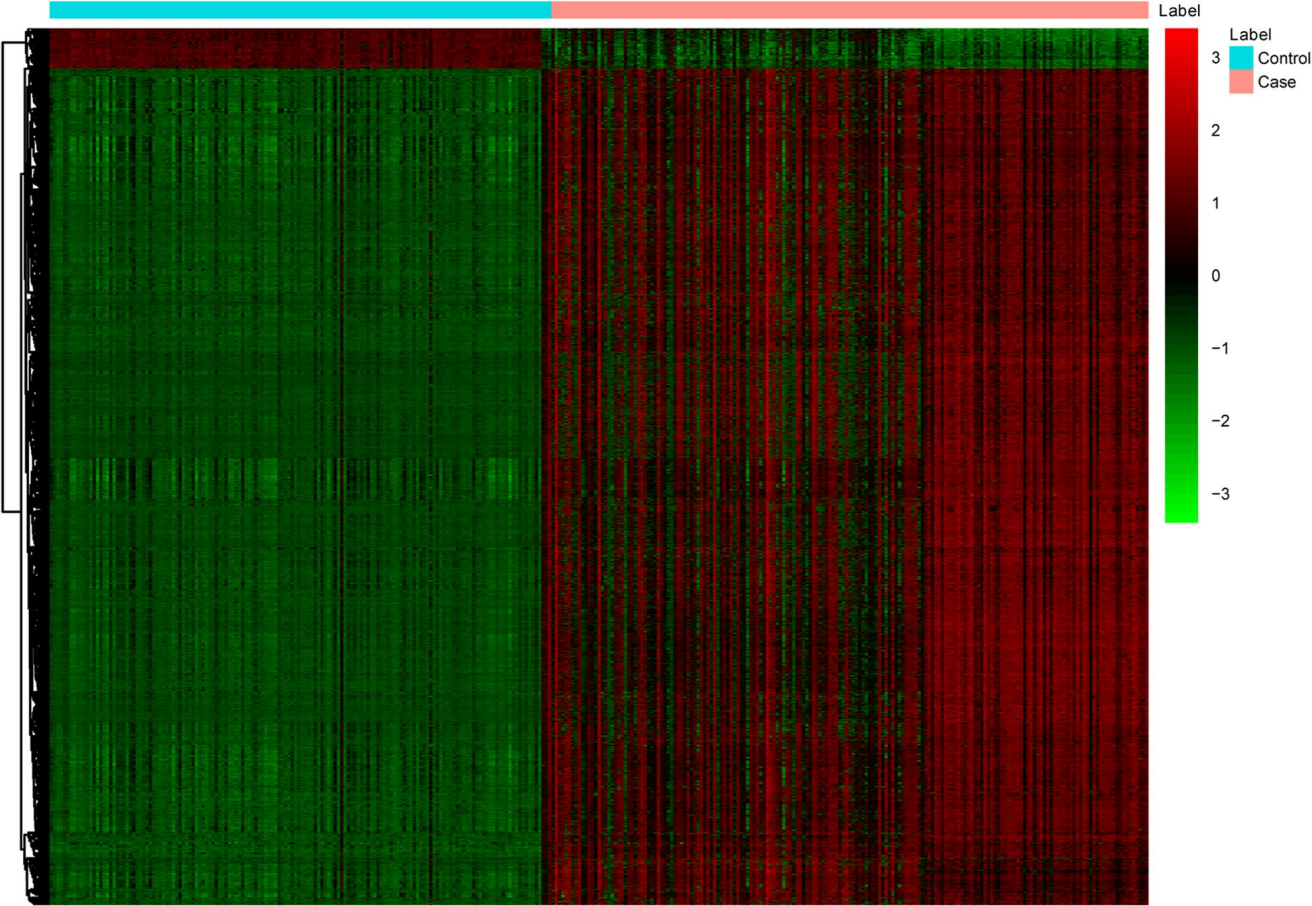
The heat map of all differentially methylated sites in CRC. Diagram presents the result of a two-way hierarchical clustering of all differentially methylated sites and samples. Each row and each column represents a differentially methylated site and a sample, respectively

There were 17,323 common genes in 3 datasets of gene transcriptome data. After scale standardization and differential expression analysis, a total of 1958 differentially expressed genes were identified in CRC. Top 20 differentially expressed genes were listed in Table 3. The heat map of top 100 differentially expressed genes was shown in Fig. 1. Additionally, there were 485,511 common methylation sites in 2 datasets of genome-wide DNA methylation expression data. After quantile standardization and differential methylation analysis, a total of 2661 differentially methylated sites were screened out in CRC. Correspondingly, there were 858 differentially methylated genes (800 hypermethylated genes and 58 hypomethylated genes) in these differentially methylated sites. The Manhattan and heat map of all differential methylated sites was shown in Fig. 2 and Fig. 3, respectively. Some differentially expressed genes, such as down-regulated *GFRA2* was hypermethylated gene. Up-regulated *MDFI* was hypomethylated gene.

**Table 3:** Top 20 differentially expressed genes in CRC

ID	Symbol	Combined.ES	***P*** value	FDR	Up/Down
94234	*FOXQ1*	4.176557	<0.05	<0.05	Up
144501	*KRT80*	4.119788	<0.05	<0.05	Up
1001	*CDH3*	3.932314	<0.05	<0.05	Up
9076	*CLDN1*	3.90363	<0.05	<0.05	Up
7472	*WNT2*	3.716528	<0.05	<0.05	Up
2118	*ETV4*	3.609427	<0.05	<0.05	Up
253152	*EPHX4*	3.577985	<0.05	<0.05	Up
84962	*AJUBA*	3.506694	<0.05	<0.05	Up
3624	*INHBA*	3.443254	<0.05	<0.05	Up
11082	*ESM1*	3.39956	<0.05	<0.05	Up
766	*CA7*	-3.36508	<0.05	<0.05	Down
10590	*SCGN*	-3.33078	<0.05	<0.05	Down
443	*ASPA*	-3.19292	<0.05	<0.05	Down
266675	*BEST4*	-3.12311	<0.05	<0.05	Down
1412	*CRYBA2*	-3.11485	<0.05	<0.05	Down
5354	*PLP1*	-3.06112	<0.05	<0.05	Down
114786	*XKR4*	-3.01472	<0.05	<0.05	Down
6387	*CXCL12*	-2.97671	<0.05	<0.05	Down
2675	*GFRA2*	-2.93584	<0.05	<0.05	Down
54738	*FEV*	-2.88959	<0.05	<0.05	Down

*ES* effect size, *FDR* false discovery rate.

### Biological function of differentially expressed and differentially methylated genes

**Fig. 4:**
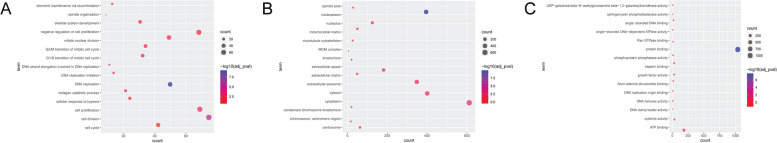
**A** Top 15 significantly enriched biological processes of differentially expressed genes. The x-axis and y-axis represents the count of differentially expressed genes and terms of biological process, respectively. **B** Top 15 significantly enriched cytological components of differentially expressed genes. The x-axis and y-axis represents the count of differentially expressed genes and terms of cytological component, respectively. **C** Top 15 significantly enriched molecular functions of differentially expressed genes. The x-axis and y-axis represents the count of differentially expressed genes and terms of molecular function, respectively

All differentially expressed genes were the most significantly enriched in the biological process of DNA replication (Fig. 4A), cytological component of nucleoplasm (Fig. 4B) and molecular function of protein binding (Fig. 4C). In addition, cell cycle, DNA replication and purine metabolism (involved *IMPDH1*) were the most remarkably enriched signaling pathways of differentially expressed genes (Table 4).

**Table 4:** The most remarkably enriched signaling pathways of differentially expressed genes

ID	Term	Count	***P*** value	Genes	FDR
hsa04110	Cell cycle	39	4.93E-09	*E2F1, E2F3, CDC14A, TTK, PRKDC, PTTG2, CHEK1, CHEK2, CCNE1, CDC45, MCM7, TFDP2, BUB1, ORC5, ORC6, CCNA2, MYC, TFDP1, ANAPC1, CDK1, RBL1, SKP2, ESPL1, CDC20, MCM2, CDK4, CDC25C, MCM3, MCM4, CDK2, MCM6, CDC25B, CCNB1, CCND1, HDAC2, CCNB2, MAD2L1, PLK1, BUB1B*	6.54E-06
hsa03030	DNA replication	19	1.10E-08	*SSBP1, LIG1, POLA1, MCM2, RNASEH2A, MCM3, MCM4, RNASEH2B, MCM6, PRIM1, POLD4, RFC3, RFC4, MCM7, RFC2, POLD1, PRIM2, POLD2, FEN1*	1.46E-05
hsa00230	Purine metabolism	40	2.78E-05	*ADCY3, XDH, ADCY5, PNPT1, POLA1, POLR2D, HPRT1, PPAT, CANT1, PDE6A, PRIM1, NUDT9, ENTPD8, PRIM2, ENTPD5, ENTPD3, PDE8A, PRPS1L1, TWISTNB, IMPDH1, PAPSS2, NUDT16, ADSSL1, POLR1E, POLR1D, PDE3A, POLR1B, AMPD2, GMPS, GART, AMPD1, POLD4, PDE7B, ADCY9, ADK, POLD1, POLD2, PDE5A, PGM1, PAICS*	0.036956

**Fig. 5:**
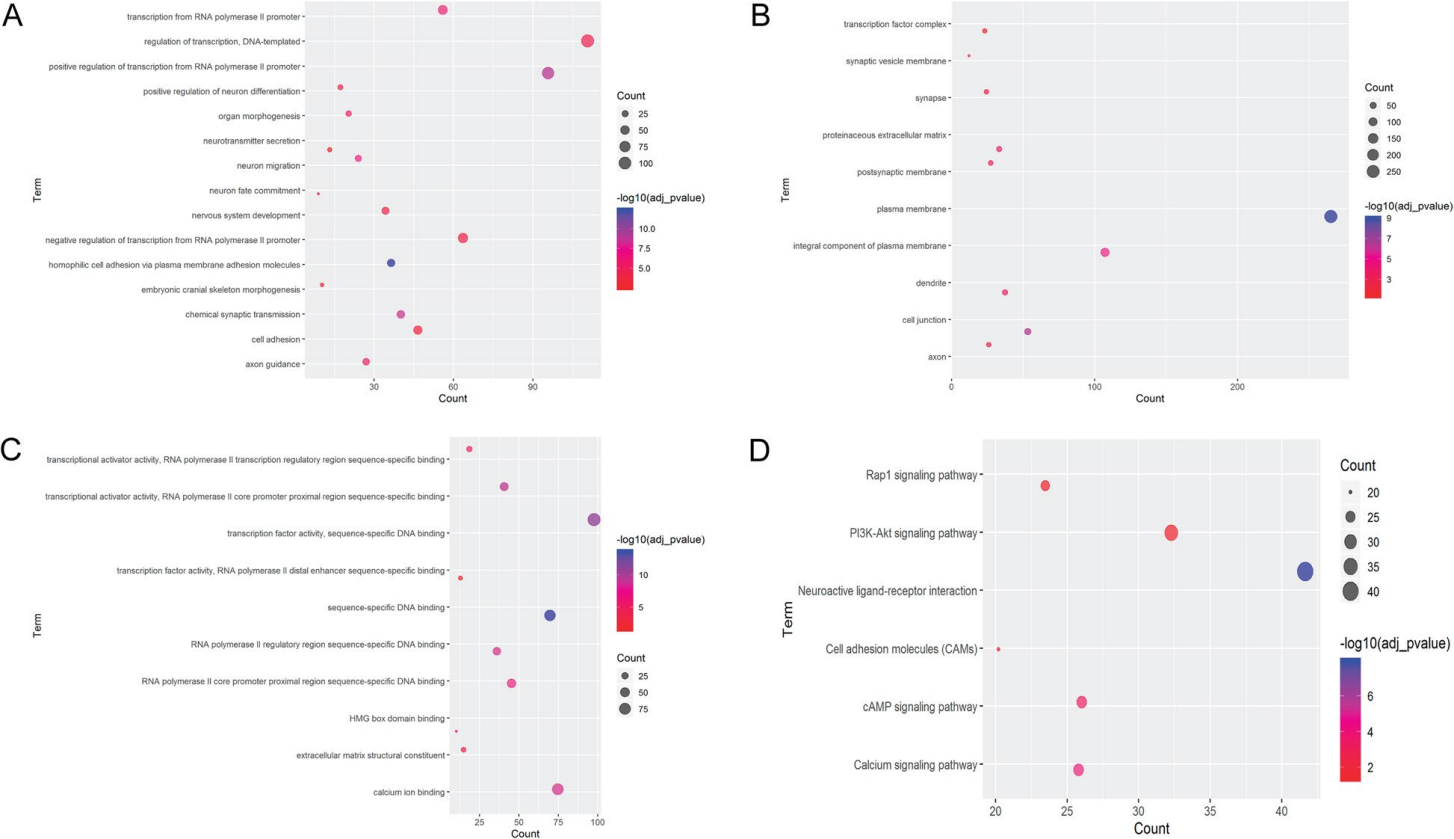
A Top 10 significantly enriched biological processes of differentially methylated genes. The x-axis and y-axis represents the count of differentially methylated genes and terms of biological process, respectively. **B** Top 10 significantly enriched cytological components of differentially methylated genes. The x-axis and y-axis represents the count of differentially methylated genes and terms of cytological component, respectively. **C** Top 10 significantly enriched molecular functions of differentially methylated genes. The x-axis and y-axis represents the count of differentially methylated genes and terms of molecular function, respectively. **D** Top 6 significantly enriched KEGG signaling pathways of differentially methylated genes. The x-axis and y-axis represents the count of differentially methylated genes and KEGG terms, respectively. The KEGG source has been obtained the permission from the Kanehisa laboratories (www.kegg.jp/feedback/copyright.html)

Additionally, all differentially methylated genes were the most significantly enriched in the biological process of homophilic cell adhesion via plasma membrane adhesion molecules (Fig. 5A), cytological component of plasma membrane (Fig. 5B) and molecular function of sequence-specific DNA binding (Fig. 5C). Neuroactive ligand-receptor interaction, calcium signaling pathway, cAMP signaling pathway, cell adhesion molecules (CAMs), PI3K-Akt and Rap1 were the most remarkably enriched KEGG signaling pathways of all differentially methylated genes (Fig. 5D).

### PPI network

**Fig. 6:**
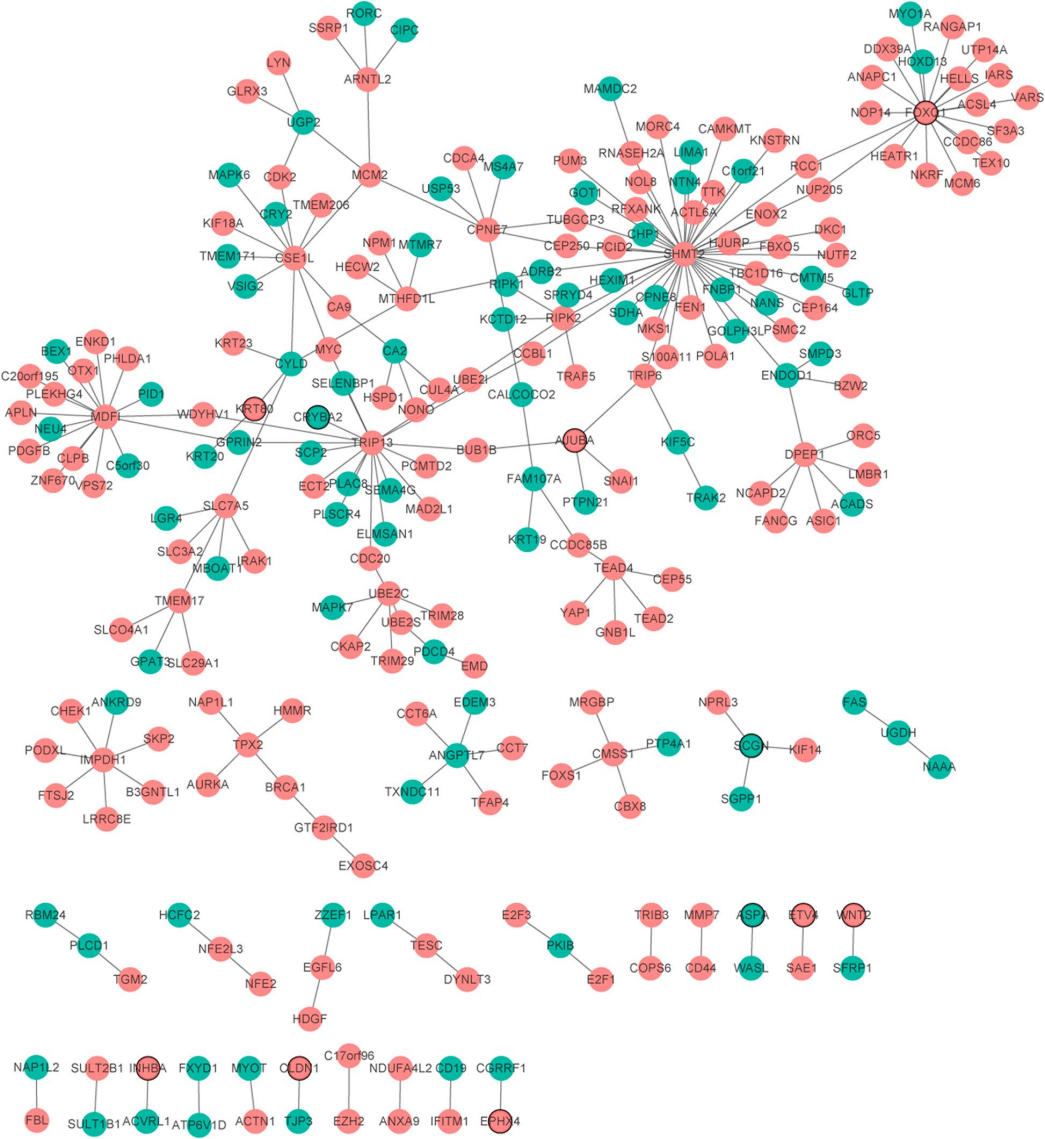
PPI networks. The red and green colors represent up-regulated and down-regulated genes, respectively. Circular with black border represent top 10 up-regulated and down-regulated genes, respectively

PPI networks of top 100 differentially expressed genes were shown in Fig. 6. The top 10 proteins with a high degree (interaction with other proteins) were *SHMT2* (degree = 44, up-regulation), *FOXQ1* (degree = 19, up-regulation), *TRIP13* (degree = 17, up-regulation), *MDFI* (degree = 16, up-regulation), *CSE1L* (degree = 11, up-regulation), *DPEP1* (degree = 7, up-regulation), *CPNE7* (degree = 7, up-regulation), *IMPDH1* (degree = 7, up-regulation), *UBE2C* (degree = 6, up-regulation) and *SLC7A5* (degree = 6, up-regulation).

### Expression validation of differentially expressed and differentially methylated genes

**Fig. 7:**
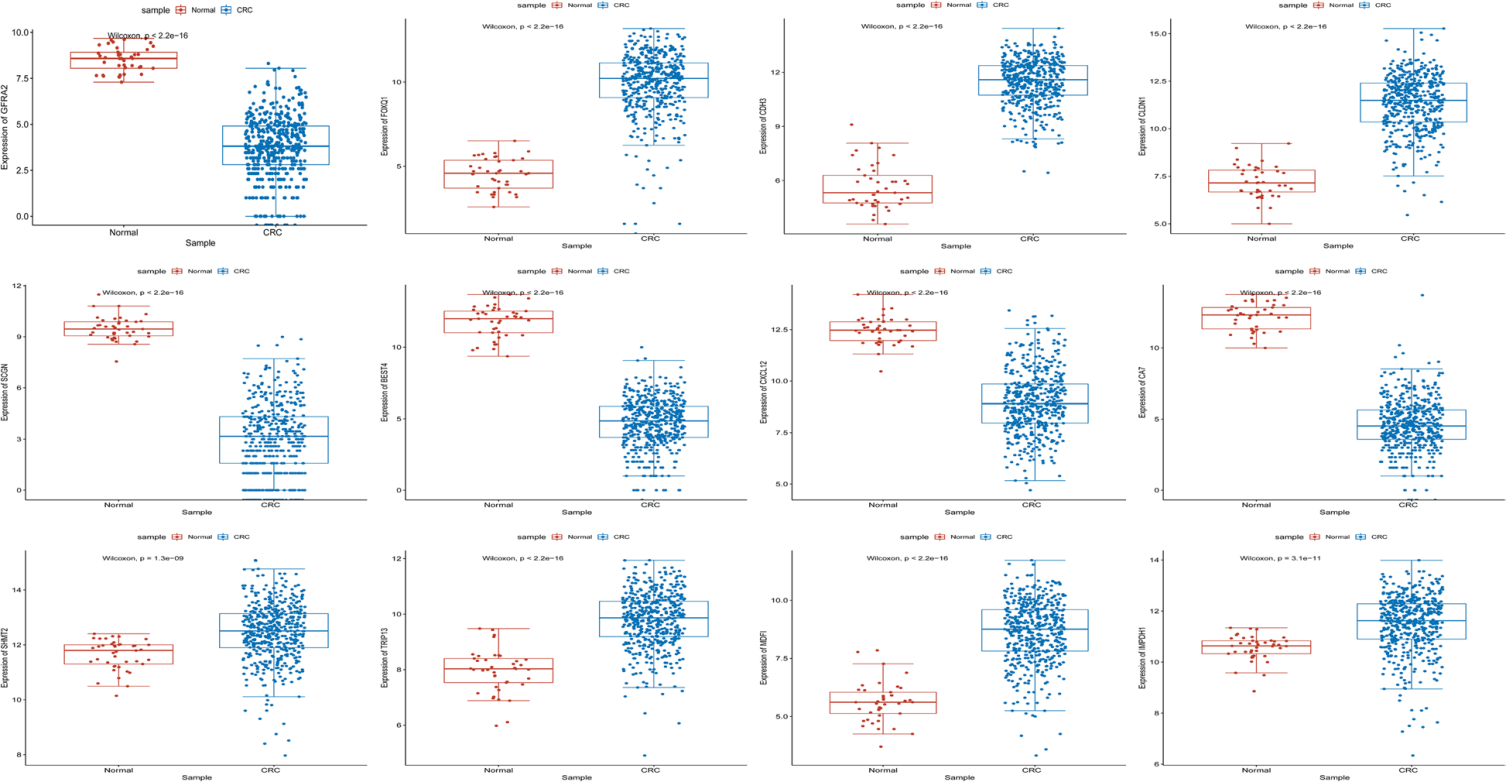
Expression box plots of *GFRA2*, *FOXQ1*, *CDH3*, *CLDN1*, *SCGN*, *BEST4*, *CXCL12*, *CA7*, *SHMT2*, *TRIP13*, *MDFI* and *IMPDH1* in the TCGA dataset

**Fig. 8:**
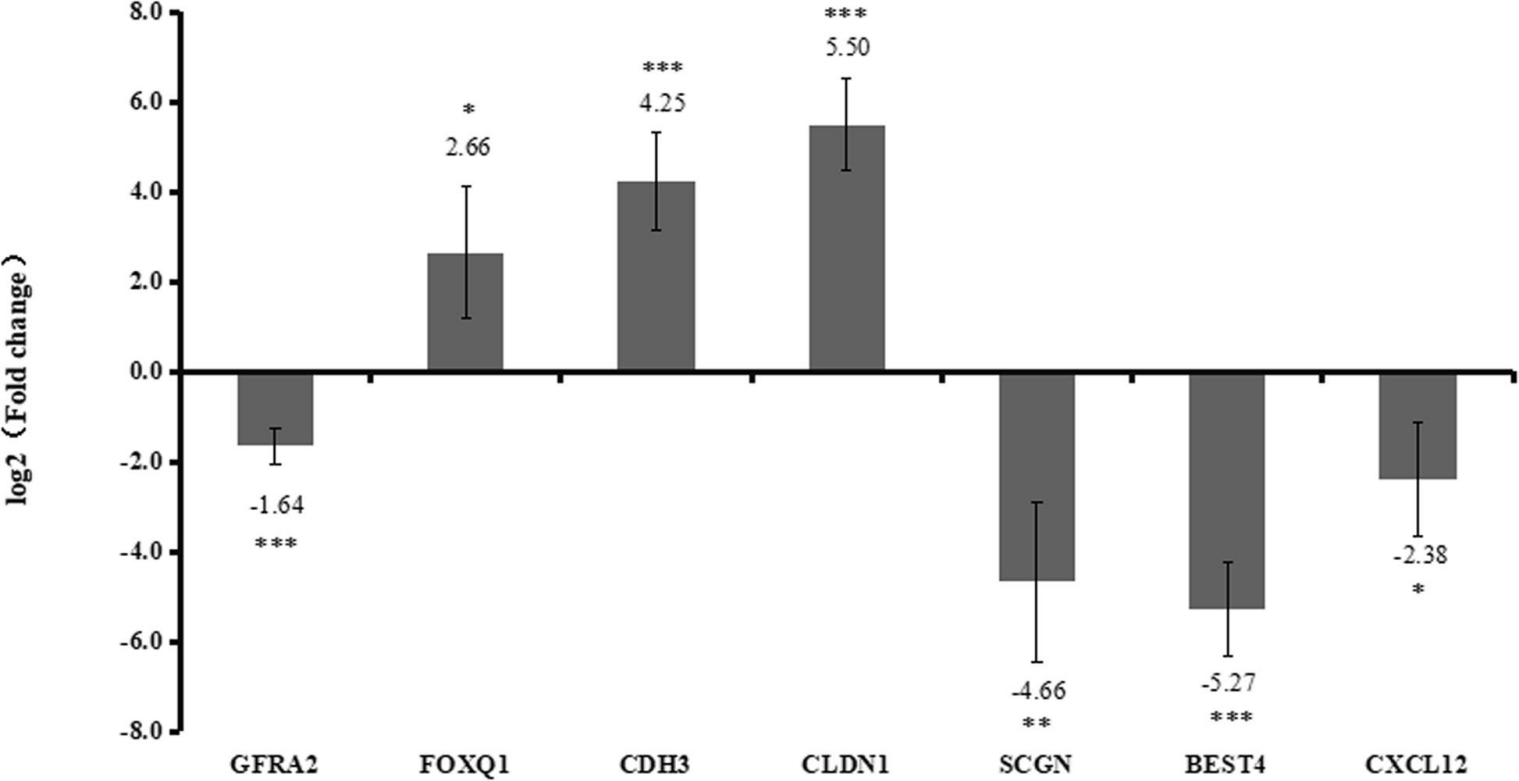
The in vitro QRT-PCR validation of *GFRA2*, *FOXQ1*, *CDH3*, *CLDN1*, *SCGN*, *BEST4* and *CXCL12* in CRC. Log2 (fold change) > 1 and log2 (fold change) < 1 represents up-regulation and down-regulation, respectively. *p < 0.05; **p < 0.01; ***p < 0.001

The TCGA dataset was firstly used to validate the expression of *GFRA2*, *FOXQ1*, *CDH3*, *CLDN1*, *SCGN*, *BEST4*, *CXCL12*, *CA7*, *SHMT2*, *TRIP13*, *MDFI* and *IMPDH1* (Fig. 7). The expression of *FOXQ1*, *CDH3*, *CLDN1*, *SHMT2*, *TRIP13*, *MDFI* and *IMPDH1* was up-regulated, while *GFRA2*, *SCGN*, *BEST4*, *CXCL12* and *CA7* were down-regulated in CRC. The in vitro experiment was applied to further validate the expression of *GFRA2*, *FOXQ1*, *CDH3*, *CLDN1*, *SCGN*, *BEST4* and *CXCL12* in 5 patients. The expression of *FOXQ1*, *CDH3* and *CLDN1* was significantly up-regulated, while the expression of *GFRA2*, *SCGN*, *BEST4* and *CXCL12* was remarkably down-regulated in CRC (Fig. 8). All the validation result was in line with the bioinformatics analysis.

### Diagnosis and survival prediction of key differentially expressed and differentially methylated genes

**Fig. 9:**
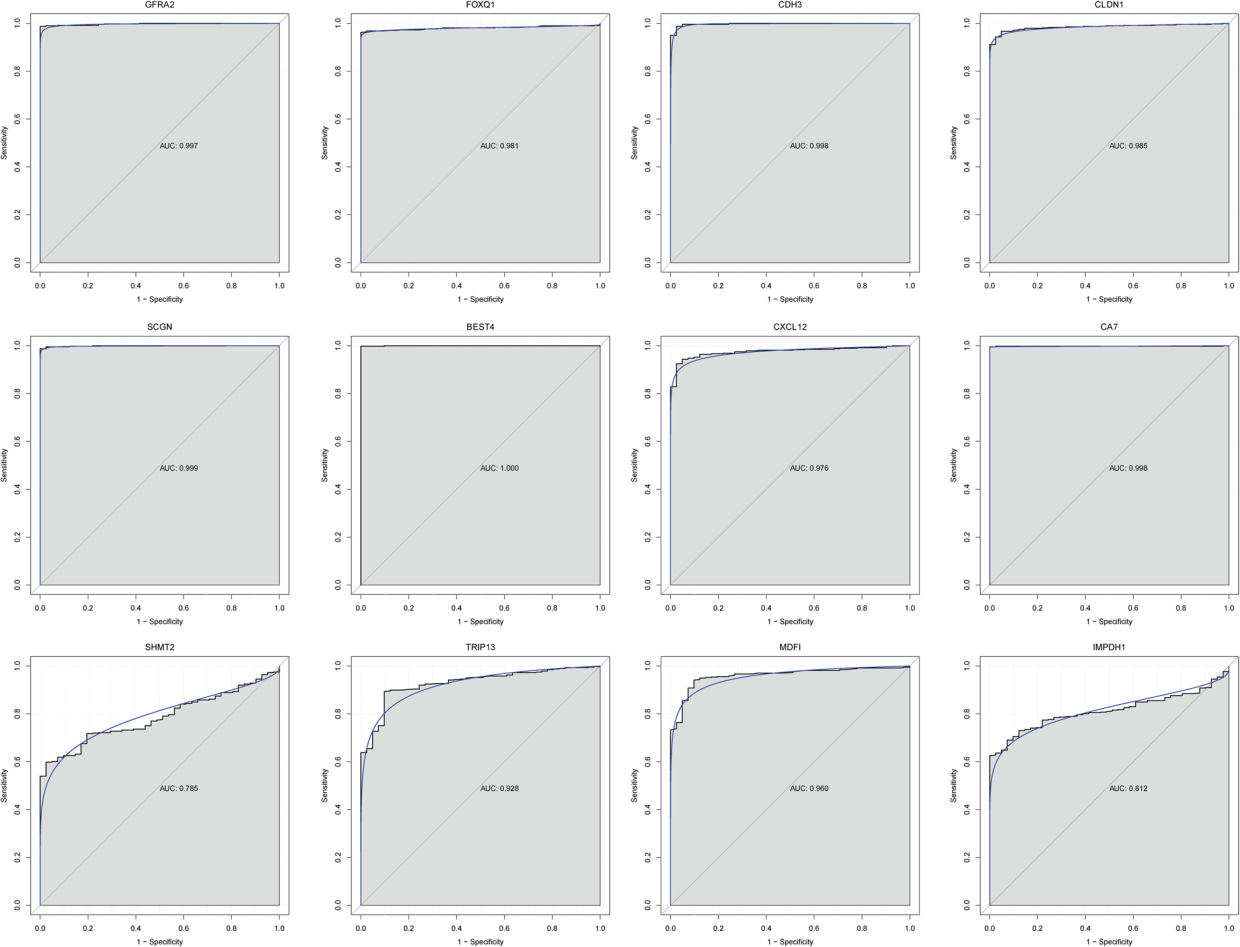
The ROC curves of *GFRA2*, *FOXQ1*, *CDH3*, *CLDN1*, *SCGN*, *BEST4*, *CXCL12*, *CA7*, *SHMT2*, *TRIP13*, *MDFI* and *IMPDH1* between CRC and normal controls. The ROC curves were used to show the diagnostic ability of these genes with 1-specificity and sensitivity

**Fig. 10:**
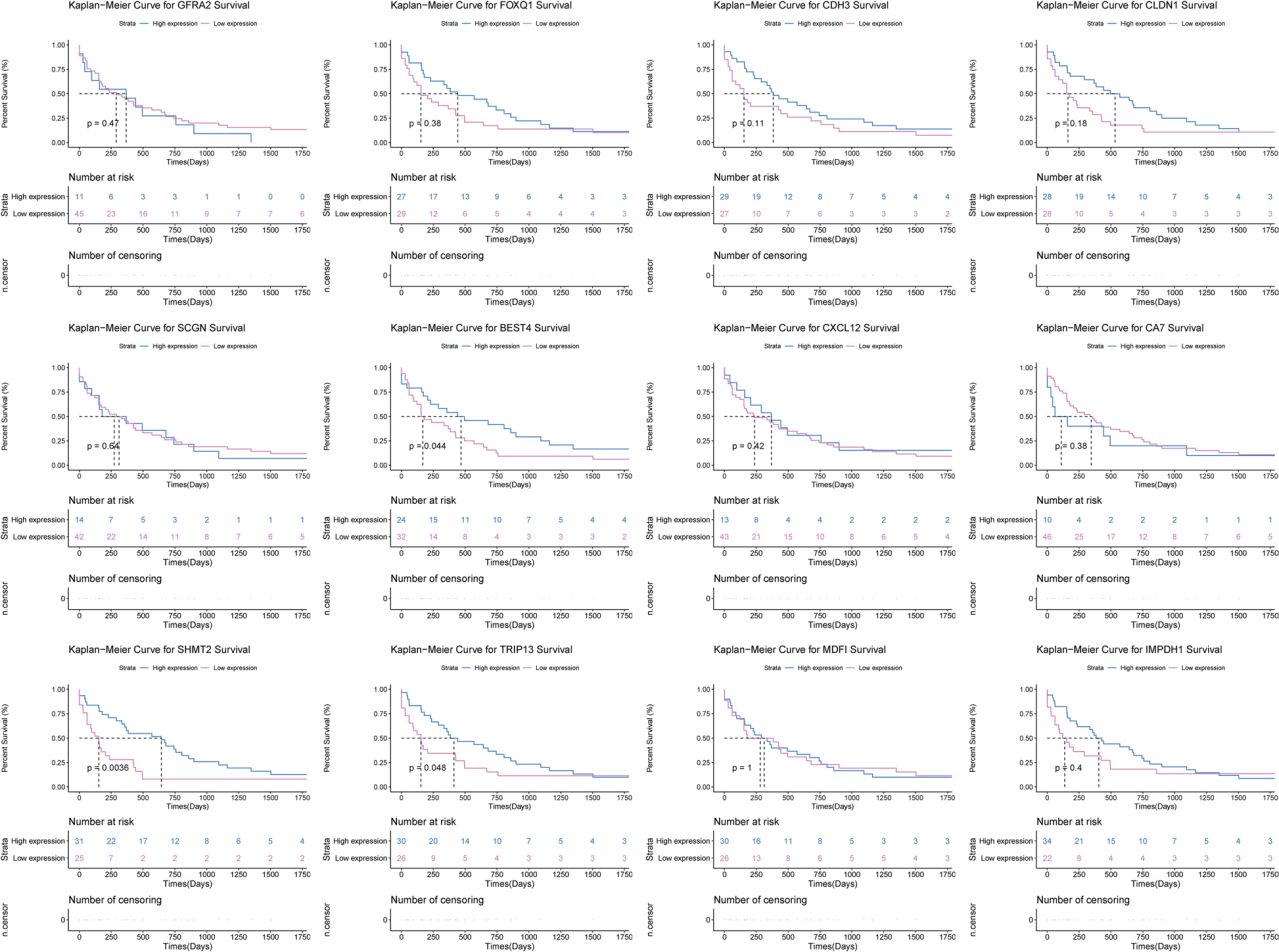
The survival curves of *GFRA2*, *FOXQ1*, *CDH3*, *CLDN1*, *SCGN*, *BEST4*, *CXCL12*, *CA7*, *SHMT2*, *TRIP13*, *MDFI* and *IMPDH1* in CRC

Firstly, we performed ROC curve analyses to assess the diagnosis ability of *GFRA2*, *FOXQ1*, *CDH3*, *CLDN1*, *SCGN*, *BEST4*, *CXCL12*, *CA7*, *SHMT2*, *TRIP13*, *MDFI* and *IMPDH1* in the TCGA dataset (Fig. 9). The AUC of these genes was more than 0.7, which suggested that they had a diagnostic value for CRC. In addition, we further analyzed the potential prognostic value of these genes. The result showed that *BEST4*, *SHMT2* and *TRIP13* were considered to be remarkably negatively associated with survival (*p* < 0.05) time with CRC patients. The survival curves of *GFRA2*, *FOXQ1*, *CDH3*, *CLDN1*, *SCGN*, *BEST4*, *CXCL12*, *CA7*, *SHMT2*, *TRIP13*, *MDFI* and *IMPDH1* were illustrated in Fig. 10.

## Discussion

GDNF family receptor alpha 2 (*GFRA2*) plays an important role in immune cells and intermediate monocytes in cancer [18, 19]. It is reported that ret. proto-oncogene (Ret) signaling through the combination of *GFRA2* and neurturin (*NRTN*) is associated with the development of enteric nervous system [20]. Macartney-Coxson DP et al. found that *GFRA2* was remarkably down-regulated in the process of CRC and possibly related to liver metastasis [21]. In mice, the function inhibition of MyoD family inhibitor (*MDFI*) promotes the regeneration of the gastrocnemius muscle after injury [22]. In addition, *MDFI* is over expressed in CRC tumors and high expression of *MDFI* is associated with tumor metastasis [22]. In this study, we found that down-regulated *GFRA2* and up-regulated *MDFI* were differentially expressed-methylated genes in CRC. This indicated that gene methylaton may be associated with gene expression changes. Moreover, *GFRA2* and *MDFI* had a diagnostic value for CRC patients. Our study further demonstrated the key roles of *GFRA2* and *MDFI* in the process of CRC.

Forkhead box Q1 (*FOXQ1*), a transcription factor, activates target mRNA expression to regulate CRC cell migration, growth, epithelial-mesenchymal transition and chemoresistance [23, 24]. It is found that *FOXQ1* is over expressed in tumor tissues of CRC and its high expression is significantly related to the stage and lymph node metastasis of CRC [25]. In addition, knock-down of *FOXQ1* gene reduces the activity of Wnt signaling pathway [25]. These reports suggest that *FOXQ1* can be considered as a potential therapeutic target for CRC. Cadherin 3 (*CDH3*), involved in cell–cell adhesion, is used to detect lymph nodes metastatic in patients with CRC [26, 27]. It has been demonstrated that hypomethylation is associated with CRC [28]. Furthermore, *CDH3* is more frequently demethylated in advanced CRC [29]. In CRC, silencing the *CDH3* genes lead to a remarkable decrease in tumor cell viability and proliferation [30]. Claudin 1 (*CLDN1*) is associated with CRC tumor invasion, lymph node metastasis and tumor grade and stage [31]. High expression of *CLDN1* has been found in primary and metastatic CRC, and CRC cell lines [32–35]. Additionally, *CLDN1* is remarkably hypomethylated in tumor samples of CRC [31]. *CLDN1* targeting with the anti-*CLDN1* monoclonal antibody reduces growth and survival of CRC cells, which suggest that *CLDN1* can be a potential new therapeutic target for CRC [36]. Herein, we found that expression *FOXQ1*, *CDH3* and *CLDN1* were top 10 up-regulated genes in CRC. Furthermore, *FOXQ1*, *CDH3* and *CLDN1* had a diagnostic value for CRC patients. Our findings may provide new insight into the cancer biology of CRC.

Secretagogin, EF-hand calcium binding protein (*SCGN*) expresses in normal endocrine tissues, such as neuroendocrine cells of gastrointestinal tract [37]. In mice, *Scgn* gene deficient leads to colitis, which highlights the role of *Scgn* in intestinal immune homeostasis [38]. The expression of bestrophin 4 (*BEST4*) is decreased in colon tumor, colon adenocarcinoma and rectal adenocarcinoma and CRC [39–42]. In addition, *BEST4* expression is remarkably negatively related to the survival probability of patients with CRC after surgery [42]. C-X-C motif chemokine ligand 12 (*CXCL12*) plays important roles in the immune system. *CXCL12* is associated with promotes CRC tumor cell growth, liver migration, survival rate and recurrence rate [43, 44]. It is reported that the *CXCL12* gene polymorphism could contribute to CRC by mediating tumor angiogenesis, progression, metastasis and leukocyte migration [45]. It is assumed that the *CXCL12-G801A* polymorphism can be used to indicate and detect stage T2 CRC [46]. In addition, activation of the CXCL12/C-X-C motif chemokine receptor 4 (*CXCR4*) axis renders CRC cell less sensitive to radiotherapy [47]. Carbonic anhydrase 7 (*CA7*) is expressed in various normal tissues including colon [48]. Decreased expression of *CA7* has been found in rectal cancer, rectal adenocarcinoma and CRC [49–51]. It is worth mentioning that CRC patients with lower *CA7* expression had a remarkable shorter disease-specific survival in early stage tumors [51]. In the present study, we found that *SCGN*, *BEST4*, *CXCL12* and *CA7* were top 10 down-regulated genes in CRC. Both of them had a diagnostic value for patients with CRC. Interestingly, *BEST4* was significantly related to survival time of CRC patients. Our result indicated that *SCGN*, *BEST4*, *CXCL12* and *CA7* could be involved in the development of CRC.

According to the PPI analysis, we found several high degree proteins encoded by differentially expressed genes, such as serine hydroxymethyltransferase 2 (*SHMT2*) and thyroid hormone receptor interactor 13 (*TRIP13*). *SHMT2*, a key regulator in the serine/glycine metabolism pathway, is involved in cancer proliferation [52, 53]. It is revealed that *SHMT2* is up-regulated in colon cancer [54]. It is noted that *SHMT2* is associated with the occurrence and development of CRC [55]. Moreover, *SHMT2* regulation by acetylation plays a crucial role in colorectal carcinogenesis [56]. *TRIP13* promotes CRC cell growth, proliferation, invasion, migration and subcutaneous tumor formation [57]. It is found that high expression of *TRIP13* is related to poor prognosis in CRC [57]. Additionally, *TRIP13* is involved in colorectal adenoma-to-carcinoma progression [58]. In our study, the expression of *SHMT2* and *TRIP13* was increased in CRC. Significantly, both *SHMT2* and *TRIP13* had a remarkable diagnostic and prognostic value for CRC.

In addition, we found some significantly enriched signaling pathways of identified genes, including purine metabolism (involved up-regulated inosine monophosphate dehydrogenase 1, *IMPDH1*), cell adhesion molecules and PI3K-Akt signaling pathway. Spurr IB et al. found that the targeting of de novo purine metabolism was a viable strategy to block tumor growth in dividing cancer cells [59]. It has been demonstrated that purine metabolism is associated with the tumorigenesis of CRC [60]. The over expression of *IMPDH1* has been found in CRC [61]. Some cell adhesion molecules such as selectins and immunoglobulin superfamily proteins play necessary roles in the CRC metastasis [62]. Ngan CY and Zlobec I et al. found that some cell adhesion molecules including E-cadherin and CD44v6 were lost at the invasive front of CRC [63, 64]. The PI3K/Akt signaling pathway plays an important role in CRC and inhibition of the pathway is a potential therapeutic strategy of CRC [65, 66].

## Conclusions

In summary, we have obtained numerous differentially expressed and differentially methylated genes in CRC. Among which, *GFRA2* and *MDFI*, were differentially expressed-methylated genes. It is suggested that DNA methylation may affect the expression changes of gene. Interestingly, *GFRA2*, *FOXQ1*, *CDH3*, *CLDN1*, *SCGN*, *BEST4*, *CXCL12*, *CA7*, *SHMT2*, *TRIP13*, *MDFI* and *IMPDH1* were considered as the potential diagnostic biomarkers for CRC. In addition, *BEST4*, *SHMT2* and *TRIP13* could be used for prognostic detection molecule in CRC patients. However, there are limitations to our study. Firstly, the larger numbers of samples are further needed; Secondly, pyrosequencing and the QRT-PCR of gene methylation are further needed to respectively validate the methylation status and investigate the expression changes of methylated genes. Thirdly, the deeper mechanism study of the CRC is also explored.

## Supplementary Information

**Additional file 1: Supplementary Table 1.** Clinical information of included datasets.

## Data Availability

All data generated or analysed during this study are publicly available from GSE113513 (https://www.ncbi.nlm.nih.gov/geo/query/acc.cgi?acc=GSE113513), GSE87211 (https://www.ncbi.nlm.nih.gov/geo/query/acc.cgi?acc=GSE87211), GSE89076 (https://www.ncbi.nlm.nih.gov/geo/query/acc.cgi?acc=GSE89076), GSE101764 (https://www.ncbi.nlm.nih.gov/geo/query/acc.cgi?acc=GSE101764) and GSE129364 (https://www.ncbi.nlm.nih.gov/geo/query/acc.cgi?acc=GSE129364).

## Abbreviations

BEST4: Bestrophin 4
CDH3: Cadherin 3
CA7: Carbonic anhydrase 7
CAMs: Cell adhesion molecules
CLDN1: Claudin 1
CRC: Colorectal cancer
CXCL12: C-X-C motif chemokine ligand 12
CXCR4: CXCL12/C-X-C motif chemokine receptor 4
FDR: False discovery rate
FOXQ1: Forkhead box Q1
GFRA2: GDNF family receptor alpha 2
GO: Gene Ontology
KEGG: Kyoto Encyclopedia of Genes and Genomes
MDFI: MyoD family inhibitor
NRTN: Neurturin
PPI: Protein-protein interaction
SCGN: Secretagogin, EF-hand calcium binding protein
SEPT9: Septin 9
SHMT2: Serine hydroxymethyltransferase 2
TCGA: The Cancer Genome Atlas
TRIP13: Thyroid hormone receptor interactor 13
