# Supplementary material for: Uncovering potential genes in colorectal cancer based on integrated and DNA methylation analysis in the gene expression omnibus database
Source: BMC Cancer. 2022 Feb 3;22:138. doi: 10.1186/s12885-022-09185-0 (PMC8815138; doi:10.1186/s12885-022-09185-0)
Supplement: Supplementary file 1 — Additional file 1: Supplementary Table 1. Clinical information of included datasets. [file 12885_2022_9185_MOESM1_ESM.docx]

**Supplementary Table 1 Clinical information of included datasets**

| **Methylation data** | **Totality** | **NC** | **CRC** |
| --- | --- | --- | --- |
| **Gender** | 333 | 152 | 181 |
| Male | 209 | 98 | 111 |
| Female | 124 | 54 | 70 |
| **Age** | 63.8 | 63.36 | 64.17 |
| <40 | 14 | 6 | 8 |
| 40~60 | 96 | 46 | 50 |
| 60-80 | 197 | 89 | 108 |
| >=80 | 26 | 11 | 15 |
| **MRNA data** |  |  |  |
| **Gender** | 443 | 199 | 244 |
| Male | 287 | 125 | 162 |
| Female | 156 | 74 | 82 |
| **Age** | 64 | 64.23 | 63.82 |
| <40 | 7 | 3 | 4 |
| 40~60 | 145 | 65 | 80 |
| 60-80 | 260 | 116 | 144 |
| >=80 | 25 | 12 | 13 |

NC: normal controls; CRC: colorectal cancer.
